# Robotic Valve Turning with a Wheeled Mobile Manipulator via Hybrid Passive/Active Compliance

**DOI:** 10.3390/s24175559

**Published:** 2024-08-28

**Authors:** Hongjun Xing, Liang Ding, Jinbao Chen, Haibo Gao, Zongquan Deng

**Affiliations:** 1 National Key Laboratory of Aerospace Mechanism, Nanjing University of Aeronautics and Astronautics, Nanjing 210016, China; chenjbao@nuaa.edu.cn; 2 State Key Laboratory of Robotics and System, Harbin Institute of Technology, Harbin 150001, China; liangding@hit.edu.cn (L.D.); gaohaibo@hit.edu.cn (H.G.); dengzq@hit.edu.cn (Z.D.)

**Keywords:** wheeled mobile manipulator, compliant end-effector, passivity-based force tracking control, hybrid passive/active compliance, robotic valve turning

## Abstract

This paper addresses the problems of valve-turning operation in rescue environments where a wheeled mobile manipulator (WMM) is employed, including the possible occurrence of large internal forces. Rather than attempting to obtain the exact position of the valve, this paper presents a solution to two main problems in robotic valve-turning operations: the radial position deviation between the rotation axes of the tool and the valve handle, which may cause large radial forces, and the possible axial displacement of the valve handle as the valve turns, which may lead to large axial forces. For the former problem, we designed a compliant end-effector with a tolerance of approximately 3.5° (angle) and 9.7 mm (position), and provided a hybrid passive/active compliance method. For the latter problem, a passivity-based force tracking algorithm was employed. Combining the custom-built compliant end-effector and the passivity-based control method can significantly reduce both the radial and the axial forces. Additionally, for valves with different installation types and WMMs with different configurations, we analyzed the minimum required number of actuators for valve turning. Simulation and experimental results are presented to show the effectiveness of the proposed approach.

## 1. Introduction

Disaster response has been an impactful research area for improving the capabilities of rescue robots. The lack of favorable disaster-response preparation, especially in critical industrial bases such as nuclear power stations, causes severe danger. For example, the 2011 Fukushima Daiichi nuclear meltdown caused by an unexpected earthquake and an accompanying tsunami, produced a large number of atomic leakages. In this incident, owing to the inadequate disaster-response preparation, many robots could not function well in this extreme environment, which significantly reduced the rescue speed [1,2].

Valves are widely used in nuclear power plants. Opening/closing valves in case of an emergency can considerably reduce the damage caused by a nuclear accident. Mobile manipulators are effective in preventing human operators from being harmed by radiation and improving operation efficiency. The DARPA Robotics Challenge (DRC), which was launched in 2012, presented a new frontier for disaster-response tasks, and valve turning was one of the eight manipulation projects [3]. Valve turning plays an indispensable role in the post-disaster rescue, and the use of mobile manipulators in this application has received considerable attention.

Mobile manipulators have been used in many scenarios and, according to the terrestrial environment, different types of locomotion are employed, including tracked, legged, and wheeled locomotion [4,5,6]. Wheeled mobile manipulators (WMMs) have promising application prospects for rescue operations owing to their high mobility and obstacle-climbing ability on flat ground. Liu et al. [7] proposed a multiple-working mode control strategy for door-opening service using a WMM, and found that by switching selected joints of the robot to work in a passive mode, the occurrence of large internal forces could be prevented. Xing et al. [8] proposed a novel approach for WMMs to simultaneously improve the force exertion capability and end-effector trajectory tracking accuracy via kinematic reconfiguration. Ding et al. [9] designed a constraint estimation method for door-opening using a WMM to achieve compliance in the case where a robot grasps a door handle in a rescue environment. Chen and Ro [10] provided an adjustment method for three admittance parameters considering human intention adaptation and system passivity for ensuring an intuitive and safe human–robot interaction with a seven-degree-of-freedom (7-DOF) manipulator.

Robotic valve turning has been widely investigated, but few studies have focused on reducing the interaction force between the end-effector and the valve handle. Ahmadzadeh et al. [11,12] designed an imitation learning method and a hybrid force/motion controller for detecting the valve position and turning the valve, respectively. Peternel et al. [13] provided a method for human and robot in cooperative execution of bolt/valve turning. There are two main approaches for the valve turning task in the DRC: single-arm manipulation [3,14] and dual-arm manipulation [15,16]. The maximum force that valve turning requires in the competition is only approximately 22.4 N [3], and most teams focused on identifying the position of the target valve.

Two main methods have been proposed for eliminating or reducing the interaction forces between the end-effector and the target object: (1) designing passive compliant mechanisms and (2) adopting active interaction control algorithms. The remote center compliance device, which was designed at the Draper Laboratory by Whitney et al. [17], is a very effective compliant mechanism that can reduce undesirable forces and torques by introducing additional compliance in the coordinate directions. There are different types of compliant end-effectors, such as underactuated hands or mechanisms enwrapped with springs or other cushion materials [18,19] and soft grippers [20]. Variable Impedance Actuators (VIAs) and Variable Stiffness Actuators (VSAs) have received increasing attention due to many novel applications involving interactions with unknown or dynamic environments in recent years [21,22,23]. However, these actuators are most compliant in a single direction, and require the participation of multiple sensors, which are not feasible for the valve turning in a rescue environment. Active interaction control can be divided into direct and indirect force control. Impedance control and hybrid force/position control are two fundamental indirect force control methods [24,25,26] that have been applied in robotic assembly, multi-robot operation systems, human–machine collaborative operation [27,28,29], etc. However, some basic problems remained with these controllers. For example, the surface geometry and the contact properties need to be known a priori for these controllers to apply desired forces accurately since they do not take into account the sensed external forces [30,31,32]. The direct force control method is able to exchange contact forces accurately and thus directly manipulate objects or apply forces on surfaces, which does not need the exact environment model [33].

Additionally, artificial intelligence and computer vision have also been employed in robotic valve turning to enhance mission success and alleviate operator pressure. Ahmadzadeh et al. [34] developed a Reactive Fuzzy Decision Maker (RFDM) to respond to the relative movement between a valve and an underwater vehicle, adjusting the robot’s movement accordingly. They also designed an apprenticeship learning method to tune the RFDM based on expert knowledge. Bai et al. [35] proposed an automatic valve turning control strategy based on teaching-learning, which incorporates teaching, model learning, and task repetition. Nishikawa and Islam implemented and evaluated an image-guided visual tracking system for autonomous grasping and manipulation in valve turning applications [36,37].

In the literature, WMMs have already been applied for compliant valve turning. However, there are few studies on the specific movement of valve-handle rotation for reducing the interaction forces between the end-effector and the valve handle, which is very important for turning a valve in a rescue environment owing to the low-precision positioning system of the mobile platform and the possible large valve resistance torque. Traditional active and passive compliance does not address all the challenges of valve turning. Although studies have been performed on human–robot interaction using hybrid active/passive compliance [38,39,40], the objective was extending the frequency range of the robotic system or augmenting the end-effector bandwidth, rather than reducing the interaction forces caused by position errors.

This paper proposes a hybrid passive/active approach to the valve-turning operation with a WMM. The main contributions of this paper are as follows: (1) the design of a novel compliant end-effector and a detailed tolerance analysis, covering both angle and position tolerances; (2) a passivity-based force tracking method for robotic valve turning, successfully extended from manipulators to WMMs; (3) a hybrid passive/active compliant control method in robotic valve-turning operation for addressing radial position errors and axial handle displacement; and (4) simulations and experiments that demonstrate the efficiency of the proposed method, rigorously supporting the mathematical conclusions on the target compliant behavior.

The remainder of this paper is organized as follows. In Section 2, the problems in robotic valve turning are explained, and compliance strategies are proposed. In Section 3, the design of a novel compliant end-effector is introduced, and tolerance analysis is performed. The model of the WMM, the active compliance method, and the hybrid passive/active compliance method for valve turning are presented in Section 4. Simulations and experiments that demonstrate the validity and performance of the proposed method are presented in Section 5. Section 6 concludes the paper.

## 2. Robotic Valve Turning Analysis and Compliance Strategies

The focus of this study is compliant robotic valve turning. The position and size of the valve handle are assumed to be determined by the vision system with limited accuracy. A significant step is compliant control of the manipulator for reducing undesirable interaction forces during the turning process. Only axial turning torque is required for valve-turning operation; all other forces/torques should be limited within the allowable ranges, such as the radial interaction force and axial force.

**Figure 1:**
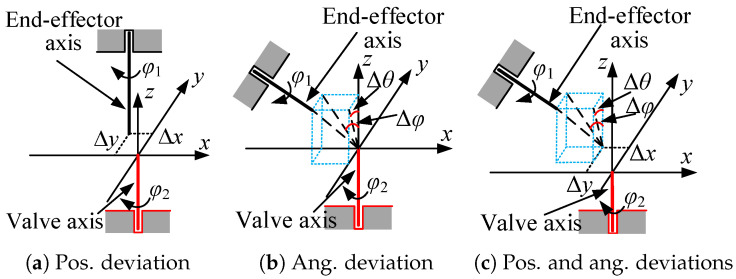
Representative misalignment scenarios between the end-effector and the valve handle.

Undesirable radial interaction force is typically caused by a misalignment between the axes of the end-effector and the valve handle, mainly due to the limited resolution of the robot vision system and the positioning inaccuracy of the mobile platform. Three representative scenarios of pose misalignment are shown in Figure 1.

In Figure 1, $\varphi_{1}$ and $\varphi_{2}$ represent the rotation angles of the end-effector and the valve handle, respectively, and Δx(Δy) and Δφ(Δθ) represent the position and angular deviations between the end-effector axis and the handle axis, respectively. In Figure 1a, there are only position deviations; in Figure 1b, there are only angular deviations; and in Figure 1c, there are both position and angular deviations.

An understanding of valve types is a prerequisite for valve turning. There are two main types of valves: rising-stem and non-rising-stem. The significant difference is that the handle of rising-stem valve moves axially with its rotation, similar to spiral motion, whereas that of the non-rising-stem valve is motionless axially. To develop a complete solution, these two main types of valves should both be considered.

According to the preceding discussions, the problems associated with robotic valve turning can be summarized as follows:

(A) The radial deviation between the end-effector and the valve handle;
(B) The possible axial displacement of the valve handle.

Small angular deviations do not cause large external forces, an shown in Figure 1b, because the end-effector can have perfect contact with the handle spokes. Additionally, big angular deviations can be avoided by an accurate vision system. Therefore, the key to solve problem (A) is to eliminate radial position deviation, as shown in Figure 1a,c, and the key to solve problem (B) is to make the end-effector track the axial position of the valve handle. Two approaches can be employed for solving the problems mentioned above (active compliance and passive compliance), and their effectiveness for valve turning is presented in Table 1.

In Table 1, Δz and Δψ represent the axial position and angular deviations, respectively, and the other symbols are defined in Figure 1. Regarding the possibility of compliance combination, “✔” and “✘” indicate that the combination is possible and not possible, respectively, and “✔✔✔” means the chosen combination is more favourable. Small radial angular deviations Δφ and Δθ do not cause large radial forces due to homogeneous contact, and the axial angular deviation Δψ can be eliminated via rotation of the end-effector. According to the previous discussions, the main factors associated with excessive forces are the position deviations in each direction. Thus, the position deviations are the main focus in the following sections.

Using passive compliance to deal with the axial displacement of the valve handle is ineffective for the following reasons. First, the axial movement is possibly massive, and passive compliance can only be used to tolerate relatively small errors. Second, some valves’ resistance torques are large, which may significantly reduce the effectiveness of passive compliance for tolerating positioning errors.

Active and passive compliance can both be used to deal with radial positioning errors. However, active compliance alone is often insufficient because even a small position deviation can result in inhomogeneous contact. Additionally, passive compliance cannot deal with large position deviations, owing to its limited compliance range. It should be noted that the boundary between small and large deviations is the compliant tool’s compliance range. Therefore, the best solution is hybrid passive/active compliance, where passive compliance is used to deal with small deviations with fast response, and active compliance ensures a large workspace.

The remainder of this section is devoted to analyzing the valid configurations of WMMs for valve turning. Most valves in nuclear power plants are installed vertically or horizontally, and these configurations may require different compliance methods. In most previous studies, the mobile platform was only in charge of moving the manipulator to a given location [3,14]. However, the existing DOFs of a mobile platform can also be considered to relax the manipulator’s requirement. Thus, two approaches—based on the manipulator alone or based on both the mobile platform and the manipulator—can be used to achieve active compliance (the intervention of the mobile platform depends on the existing DOFs of the manipulator, if the manipulator has enough DOFs to conduct valve turning, then, the mobile platform will remain immobile). The analysis results for various WMM configurations and valve installation types are presented in Table 2.

In Table 2, regardless of the installation type, the fewest number of actuators is the same when identical WMM compliant elements are adopted. When active compliance based on both the platform and the manipulator is selected, owing to the different orientations of the valve handle, the WMM compliant elements are not the same for problems (A) and (B), as indicated by the last column of Table 2. When using active compliance based on the manipulator, the manipulator’s DOF handle the valve turning process, while the mobile platform facilitates system navigation. Conversely, if a mobile manipulator is employed to achieve compliance, both the DOFs of the mobile platform and the manipulator will be utilized for the valve turning operation. The next section presents the proposed design of a suitable passive compliant end-effector, and an efficient active compliance control system is presented in Section 4.

## 3. Design and Analysis of Compliant End-Effector

### 3.1. Design of Compliant End-Effector

**Figure 2:**
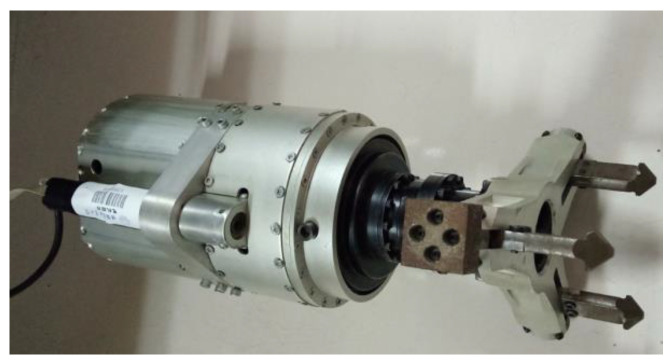
The designed compliant end-effector.

In addition to realizing passive compliance, the designed compliant end-effector can accommodate replaceable tools for rotating various valve handles via detachable design, but this is not the topic in this paper. The designed compliant end-effector (a compliant unit and a tool) is shown in Figure 2. The attached three-finger tool is used to rotate a five-spoke valve.

**Figure 3:**
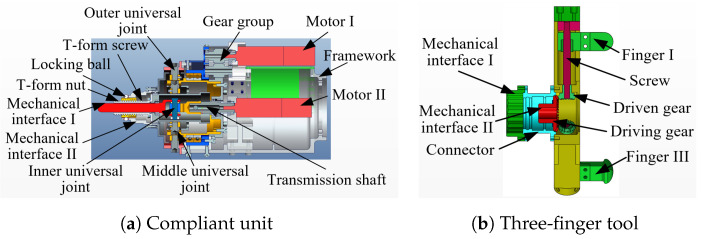
Section views of the compliant end-effector.

A section view of the compliant end-effector is shown in Figure 3.

As shown in Figure 3a, the universal joint is adopted as the compliant mechanism, and for connecting and transmitting power to the tool, a novel three parallel universal (3PU) joint mechanism is designed. It is worth noting that the rotation joints’ stiffness is minimal to accommodate the target valve. This compliant end-effector is advantageous in valve turning due to its multi-directional compliance performance with a simple structure. The power required for controlling the tool, locking the tool, and rotating the valve handle is transmitted to the tool through the inner universal joint, middle universal joint, and outer universal joint provided by Motor I, Motor II, and the last joint of the manipulator, respectively. The bevel gear and screw mechanisms are employed by the tool to realize the synchronous motion of the three fingers for changing the tool workspace, as shown in Figure 3b. Each finger has a bulge at the end to hook the handle up and can be replaced to adapt to handles with different thicknesses.

The 3PU joint plays a significant role in the compliant end-effector, and the three universal joints deflect congruously in the radial direction by adopting bearings. The maximal deflection angle, which is designed to be controlled by the outer universal joint, dominates the tolerance of the end-effector. The tolerance is defined as the maximal passive radial displacement of the tool.

### 3.2. Tolerance Analysis of Compliant End-Effector

The deflection angle of the 3PU is that of the compliant end-effector, and for tolerance analysis, the 3PU can be simplified as a single universal joint.

**Figure 4:**
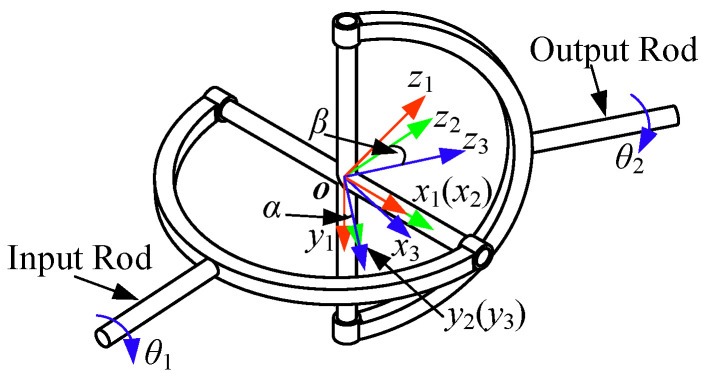
3D model of a typical universal joint.

The 3D model of a typical universal joint is shown in Figure 4. For easy description, four coordinate systems ({0}, {1}, {2}, and {3}) are established. The first one is the world coordinate system (overlapping with the initial pose of {1}). The other three coordinate systems are attached to the input rod, cross shaft, and output rod, respectively, and the origins of the four coordinate systems are at the same point. The deflection angles between {1} and {2} around $y_{1}$ and between {2} and {3} around $z_{2}$ are α and β, respectively, and $\theta_{1}$ and $\theta_{2}$ are the input and output angles of the universal joint, respectively.

**Figure 5:**
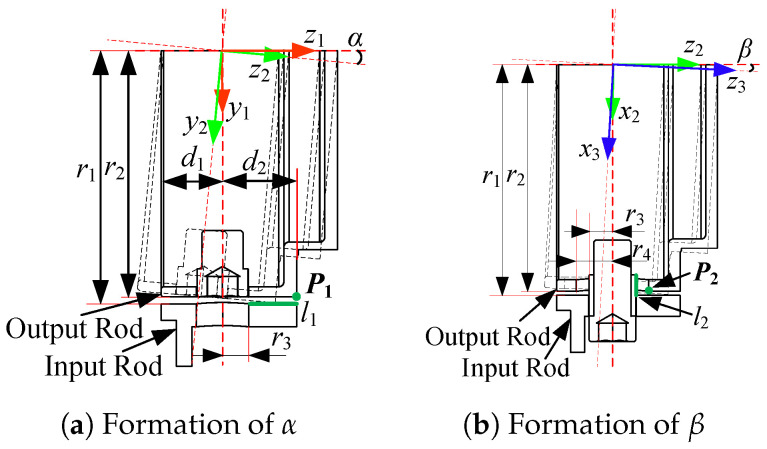
Structure of the outer universal joint.

The tolerance is mainly determined by the deflection angles α and β, which are constrained by the parallel structure. Figure 5 presents the outer universal joint. Only half of it is shown, as it is symmetrical. As shown in Figure 5a, the deflection angle α is related to the radii of the input and output rods $r_{1}$ and $r_{2}$, respectively, and the forward and backward widths $d_{2}$ and $d_{1}$, respectively. With $d_{1}<d_{2}$, the maximum of α is constrained by the contact between point $P_{1}$ of the output rod and edge $l_{1}$ of the input rod, which is defined as $\varsigma_{1}$.

Edge $l_{1}$ can be expressed in {1} as

(1) l11=[0,r1,t1]T,t1∈(r3,d2),

where $r_{3}$ is the shaft radius of the input rod, as shown in Figure 5b. Point $P_{1}$ can be expressed in {2} as

(2) P12=[0,r2,d2]T.

Point $P_{1}$ contacts edge $l_{1}$ when their coordinates in {1} overlap, i.e.,

(3) l11=Rx(α)P12,

where $R_{x(\alpha )}$ is the rotation matrix. According to (3),

(4) $$\varsigma_{1}=\alpha_{\max}=\arcsin\left(\frac{r_{1}}{\sqrt{r_{2}^{2}+d_{2}^{2}}}\right)-\varphi ,$$

where $\tan\varphi =r_{2}/d_{2}$. Considering the range of $t_{1}$ in (1), the value of $r_{3}$ should satisfy

(5) $$r_{3}<\sqrt{r_{2}^{2}+d_{2}^{2}}\cos\left(\varsigma_{1}+\varphi \right).$$

As shown in Figure 5b, the deflection angle β is not only limited by the factors, similarly to α, but also dependent on the shaft radius of the input rod $r_{3}$ and the hole radius of the output rod $r_{4}$. The design objective of β is to make its maximum value $\varsigma_{2}$ equal to $\varsigma_{1}$; thus, the collision between point $P_{2}$ of the output rod and edge $l_{2}$ of the input rod should not appear in the formation of $\varsigma_{2}$.

Under the assumption that $l_{2}$ is sufficiently large, the contact conditions of $l_{2}$ and $P_{2}$ can be obtained as

(6) $${\beta^{\prime }}_{\max}=\arccos\left(\frac{r_{3}}{\sqrt{r_{2}^{2}+r_{4}^{2}}}\right)-\phi ,$$

where $\tan\phi =r_{2}/r_{4}$. According to the inequality ${\beta^{\prime }}_{\max}⩾\varsigma_{1}$, the following requirement can be obtained through (4) and (6):

(7) $$\arccos\left(\frac{r_{3}}{\sqrt{r_{2}^{2}+r_{4}^{2}}}\right)⩾\arctan\frac{r_{2}}{r_{4}}+\varsigma_{1}.$$

When (5) and (7) are satisfied, the maxima of the deflection angles α and β are both equal to $\varsigma_{1}$.

The deviation angle between the input rod and the output rod, defined as ϑ, can be calculated using the obtained α and β. This deviation angle determines the tolerance of the end-effector.

The input and output rods in Figure 4 are defined as U1 and V3 in {1} and {3}, respectively, and V3 can be expressed in {1} as

(8) V1=Rx(α)Ry(β)V3,

where $R_{x(\alpha )}$ and $R_{y(\beta )}$ are rotation matrices. Then, we obtain

(9) ϑ=arccos(UT1V1U1V1)=arccos(cosαcosβ).

**Figure 6:**
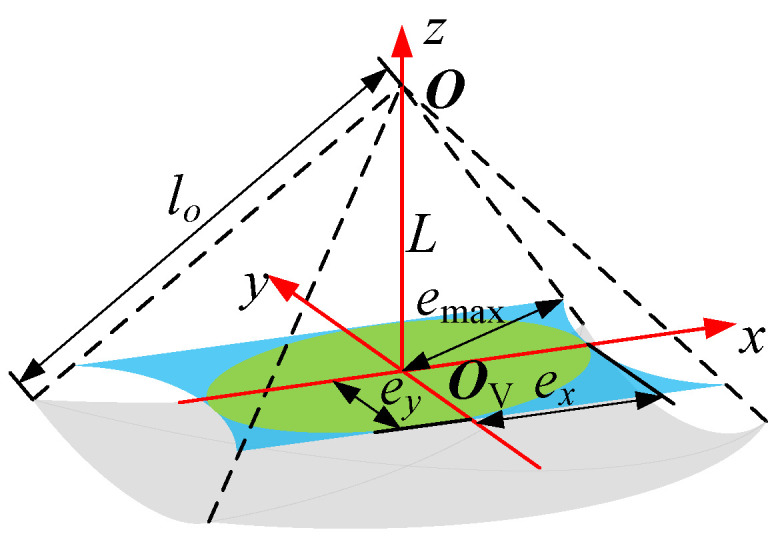
The tolerance of the end-effector (the gray region represents the position of the output rod end, the blue part represents the complete tolerance, and the green area represents the optimal tolerance).

Equation (9) gives the deviation angle between the two rods, which is maximized when both α and β reach their peaks. The tolerance of the end-effector is described in Figure 6. We define the axial distance between the universal joint center (point O in Figure 4) and the valve handle plane as *L*, where point $O_{V}$ denotes the center of the valve handle. Here, we provide a detailed explanation of the colored areas in Figure 6 to provide a clearer understanding of their meanings. The gray region is formed by the end point of the output rod when the output rod rotates ±α around $y_{1}$ and ±β around $z_{2}$.

In Figure 6, $l_{o}$ denotes the length of the output rod, the directions of *x* and *y* coincide with those of $x_{0}$ and $y_{0}$ in the world coordinate system, and the values of the parameters are

(10) $$\left\{\begin{array}{c} e_{x}=L\tan\varsigma_{1},\\ e_{y}=L\tan\varsigma_{1},\\ e_{\max}=L\tan\varsigma_{1}\sqrt{1+\cos^{2}\varsigma_{1}}/\cos\varsigma_{1}. \end{array}\right.$$

It should be noted that the valve turning is a rotation operation, while the complete tolerance is not a circle. Therefore, $e_{x}$ in Figure 6 is defined as the tolerance of the end-effector.

## 4. Hybrid Passive/Active Compliance Approach for Valve Turning

### 4.1. Kinematic and Dynamic Modeling of WMMs

A WMM is mainly composed of a wheeled mobile platform and a manipulator. Normally, the mobile platform subsystem is subjected to nonholonomic constraints, while the constraints for the manipulator are holonomic. Kinematics deal with geometric relationships without considering the role of forces, while dynamics study the behaviors caused by forces and torques.

Define the generalized coordinate vector of the WMM as $q={\left[q_{p}^{T},q_{m}^{T}\right]}^{T}$, where $q_{p}\in R^{g_{p}}$ and $q_{m}\in R^{g_{m}}$ are the generalized joint coordinate vectors of the mobile platform and the manipulator, respectively. Then, the pose of the end-effector can be expressed with respect to *q* as $x\left(q_{p},q_{m}\right)\in R^{n}$. Here, the function of *x* with respect to $q_{p}$ is obtained considering the nonholonomic constraints of the mobile platform, and *x* with respect to $q_{m}$ can be derived using D-H parameters [41].

Assuming a pure rolling contact between the wheels and the ground (i.e., no slippage), then the platform’s kinematic model with nonholonomic constraints can be derived as

(11) $${\dot{q}}_{p}=P\left(q_{p}\right)v_{p},$$

where $v_{p}\in R^{p}$ is the velocity input vector of the wheels, with $p<g_{p}$; and $P\in R^{g_{p}\times p}$ transforms wheel velocities into generalized platform velocities. It is worth noting that the skidding and slipping phenomena of the mobile platform are not analyzed in this paper, because they are not pronounced due to the massive weight of the WMM during the valve-turning operation. The research about skidding and slipping modeling of WMMs can be found in a lot of literature, including by our research group [42]. The generalized manipulator velocity vector can be expressed using joint velocities as

(12) $${\dot{q}}_{m}=v_{m},$$

where $v_{m}\in R^{m}$ contains the joint velocities, with $m=g_{m}$. Then, the velocity input vector of a WMM system can be expressed as $v={\left[v_{p}^{T},v_{m}^{T}\right]}^{T}\in R^{p+m}$.

The end-effector velocity is obtained by taking the first derivative of *x* to time. Combining (11) and (12) yields [43]

(13) $$\dot{x}=\frac{\partial x}{\partial q_{p}}{\dot{q}}_{p}+\frac{\partial x}{\partial q_{m}}{\dot{q}}_{m}=\left[J_{p}\left(q\right)P\left(q_{p}\right)\;J_{m}\left(q\right)\right]\left[\begin{array}{c} v_{p}\\ v_{m} \end{array}\right]=J\left(q\right)v.$$

where $J\left(q\right)\in R^{n\times (p+m)}$ is the Jacobian of the WMM. Thus, the total WMM kinematic model is derived as (13).

Then, the motion input vector for the WMM can be expressed as

(14) $$v={\left(J\left(q\right)\right)}^{\#}\dot{x},$$

where ${\left(J\left(q\right)\right)}^{\#}$ denotes the pseudoinverse of J(q).

Inspired by [44], the dynamic model of a WMM can be expressed as

(15) $$\left[\begin{array}{c} M_{pp}\;M_{pm}\\ M_{pm}^{T}\;M_{mm} \end{array}\right]\left[\begin{array}{c} {\dot{v}}_{p}\\ {\dot{v}}_{m} \end{array}\right]+C\left(q_{p},{\dot{q}}_{p},q_{m},{\dot{q}}_{m}\right)\left[\begin{array}{c} v_{p}\\ v_{m} \end{array}\right]+\left[\begin{array}{c} g_{p}\\ g_{m} \end{array}\right]=\left[\begin{array}{c} \tau_{p}\\ \tau_{m} \end{array}\right]+\tau_{ext},$$

where $M_{pp}\in R^{p\times p}$, $M_{pm}\in R^{p\times m}$, and $M_{mm}\in R^{m\times m}$ are the inertia matrices for the platform, the coupling between the platform and the manipulator, and the manipulator, respectively; $C\left(q_{p},{\dot{q}}_{p},q_{m},{\dot{q}}_{m}\right)\in R^{\left(p+m)\times (p+m\right)}$ represents the centrifugal and Coriolis matrix; $g_{p}\in R^{p}$ and $g_{m}\in R^{m}$ are the gravitational terms for the platform and the manipulator, respectively; $\tau_{p}\in R^{p}$ and $\tau_{m}\in R^{m}$ are the controlled torque input vector for the platform and the manipulator, respectively; and $\tau_{ext}={\left[\tau_{pext}^{T},\tau_{mext}^{T}\right]}^{T}\in R^{p+m}$ is the joint torque vector caused by the external forces/torques.

Based on (15), we make some assumptions for our research:

**Assumption 1.**  *A velocity-based platform motion controller can compensate any dynamic effects and realizes any given velocity within its available ranges.*

**Assumption 2.**  *With low velocity, the dynamics of the mobile platform can be separated from the entire WMM system’s dynamics.*

Considering (15), these assumptions mean that the dynamic effects between the platform and the manipulator are negligible. And for controller design, the manipulator is separated from the platform, which means $M_{pm}$ is omitted to decouple the two subsystems.

With consideration of a velocity-controlled mobile platform, an admittance interface is employed to apply the controlled torque input $\tau_{p}$ to the platform

(16) $$M_{v}{\dot{v}}_{p}+D_{v}v_{p}=\tau_{p},$$

where $M_{v}\in R^{p\times p}$ and $D_{v}\in R^{p\times p}$ are the desired virtual inertia and damping for the platform, respectively. It should be noted that the admittance behavior (16) does not represent the real dynamics for the platform, but only an approximate one we expect it to show.

### 4.2. Design of Active Compliance Approach

Active compliance is essential to compensate for large radial deviation and axial handle displacement. Force tracking control is a desirable approach because only an approximate position of the target valve is obtained; by tracking external forces during the operation, the multidimensional position deviation can be effectively compensated. Hence, in the remaining sections, the main objective is to limit the external forces to a permissible range. According to (13) and the virtual work principle, the end-effector forces can be mapped to joint torques via $J^{T}\left(q\right)$ as

(17) $$\tau_{ext}=J^{T}\left(q\right)F_{ext},$$

where $F_{ext}\in R^{n}$ and $\tau_{ext}\in R^{p+m}$ are the Cartesian end-effector force vector (measured by a wrist force/torque sensor) and its projected value in joint space, respectively. Inspired by [45], the following PID-type force tracking controller is adopted in this research:

(18) $$\tau =J^{T}\left(q\right)\left[K_{p}\left(F_{ext}\left(t\right)-F_{d}\left(t\right)\right)+K_{d}\left({\dot{F}}_{ext}\left(t\right)-{\dot{F}}_{d}\left(t\right)\right)-K_{i}h_{i}\left(F_{ext},t\right)\right],$$

where $h_{i}\left(F_{ext},t\right):=\int_{0}^{t}\left(F_{d}\left(\sigma \right)-F_{ext}\left(\sigma \right)\right)d\sigma$; $K_{p}\in R^{n\times n}$, $K_{d}\in R^{n\times n}$, $K_{i}\in R^{n\times n}$ are diagonal positive definite matrices for the PID controller, respectively; $\tau ={\left[\tau_{p}^{T},\tau_{m}^{T}\right]}^{T}\in R^{p+m}$ represents the controlled torque vector for the robotic system; and $F_{d}\left(t\right)\in R^{n}$ is the desired force/torque vector applied on the environment. It is worth mentioning that a fourth-order Butterworth low-pass filter should be performed on the measured force and its derivative to mitigate the measurement noise effect.

By establishing the WMM dynamic model, and applying (18) into (15), we can realize position tracking indirectly by force tracking in partly unknown environment. The stability of this controller for WMM is subsequently proven.

First, we virtually decompose the entire WMM system into three parts: environment, WMM dynamics and force tracking controller. According to [46], the entire system is passive if each subsystem is passive, no matter the blocks are connected in parallel or feedback. Since the WMM dynamics (15) is passive with respect to the input-output pair $\left[\tau +\tau_{ext},\dot{q}\right]$ and the environment is assumed to be passive with respect to $\left[\dot{q},-\tau_{ext}\right]$. So, if the force tracking controller is proven to be passive, then the entire system’s stability is guaranteed.

**Figure 7:**
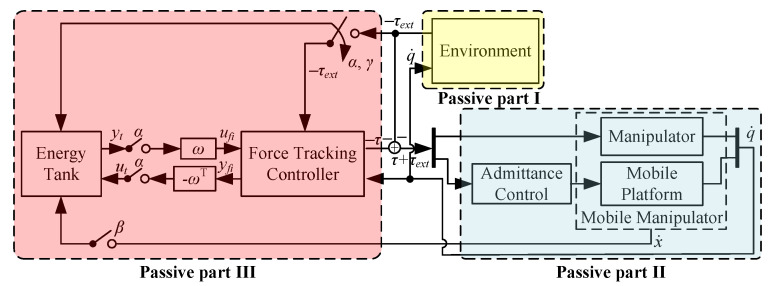
Force tracking control system for WMM.

The concept of energy tank is utilized to modify the controller to preserve its passivity [45,47]. Inspired by these, the proposed control system is shown in Figure 7.

A virtual energy tank is introduced first with its state defined as $x_{t}$ and output as $y_{t}=x_{t}$. Then, the energy for the tank is $T=x_{t}^{2}/2$ with

(19) $${\dot{x}}_{t}=\frac{\beta }{x_{t}}{\dot{x}}^{T}K_{d}\left({\dot{F}}_{d}-{\dot{F}}_{ext}\right)+u_{t},$$

where β is defined as

(20) $$\beta =\left\{\begin{array}{cc} 1, & \;\mathrm{if}\;\;T⩽T^{u}\\ 0, & \;\mathrm{else}\; \end{array}\right.$$

with $T^{u}$ denoting the upper limit of the energy storage. The input of the tank is defined as $u_{t}=-\omega^{T}\dot{x}$, with

(21) $$\omega \left(F_{ext},t\right)=\frac{\alpha }{x_{t}}(K_{p}\left(F_{ext}-F_{d}\right)+\left(1-\gamma \right)K_{d}\left({\dot{F}}_{d}-{\dot{F}}_{ext}\right)-K_{i}h_{i},$$

where α is defined as

(22) $$\alpha =\left\{\begin{array}{cc} 1, & \;\mathrm{if}\;\;T⩾T_{l}\\ 0, & \;\mathrm{else}\; \end{array}\right.$$

with $T_{l}$ denoting the lower limit of the energy storage. γ in (19) and (21) is defined as

(23) $$\gamma =\left\{\begin{array}{cc} 1, & \;\mathrm{if}\;\;{\dot{x}}^{T}K_{d}\left({\dot{F}}_{d}-{\dot{F}}_{ext}\right)⩾0\\ 0. & \;\mathrm{else}\; \end{array}\right.$$

Subsequently, we define the input of the force tracking controller to be $u_{fi}=\omega x_{t}$ and the output to be $y_{fi}=\dot{x}$. The storage function of part III is chosen as $T\left(x_{t}\right)=x_{t}^{2}/2$ with its timely evolution

(24) $$\begin{array}{c} \begin{array}{cc} \dot{T}\left(x_{t}\right)= & x_{t}{\dot{x}}_{t}=\beta \gamma K_{d}\left({\dot{F}}_{d}-{\dot{F}}_{ext}\right)\\ \; & -\alpha {\left[K_{p}\left(F_{ext}-F_{d}\right)+\left(1-\gamma \right)K_{d}\left({\dot{F}}_{d}-{\dot{F}}_{ext}\right)-K_{i}h_{i}\right]}^{T}\dot{x}. \end{array} \end{array}$$

In force tracking control, $\tau ={\left[\tau_{p}^{T},\tau_{m}^{T}\right]}^{T}$, and according to (18),

(25) $$-{\dot{q}}^{T}\tau =\underset{\approx {\dot{x}}^{T}}{\underset{︸}{{\dot{q}}^{T}J^{T}}}\left[K_{p}\left(F_{d}-F_{ext}\right)+K_{d}\left({\dot{F}}_{d}-{\dot{F}}_{ext}\right)+K_{i}h_{i}\right],$$

then, combining (24) and (25), the following conclusion can be derived:

(26) $$\dot{T}\left(x_{t}\right)=-{\dot{q}}^{T}\tau -\gamma \left(1-\beta \right){\dot{x}}^{T}K_{d}\left({\dot{F}}_{d}-{\dot{F}}_{ext}\right).$$

Since $T(x_{t})⩾0$ and obviously $\dot{T}\left(x_{t}\right)⩽-{\dot{q}}^{T}\tau$ due to (20) and (23), then part III is passive. Hence, the entire control system is stable.

### 4.3. Hybrid Passive/Active Compliance for Robotic Valve Turning

#### 4.3.1. Hybrid Passive/Active Compliance Method for Radial Position Deviation

**Figure 8:**
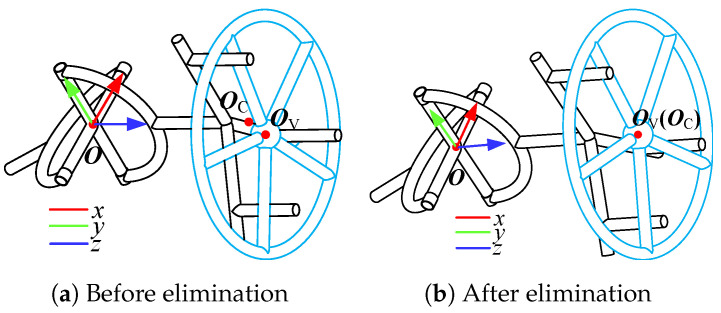
Radial deviation elimination.

The radial deviation elimination is described in Figure 8. In Figure 8a, O, $O_{C}$, and $O_{V}$ are the center of the universal joint, the intersection point of the output shaft and the handle plane, and the center of the handle plane, respectively. The objective is to make point $O_{C}$ and point $O_{V}$ coincide, as shown in Figure 8b.

**Figure 9:**
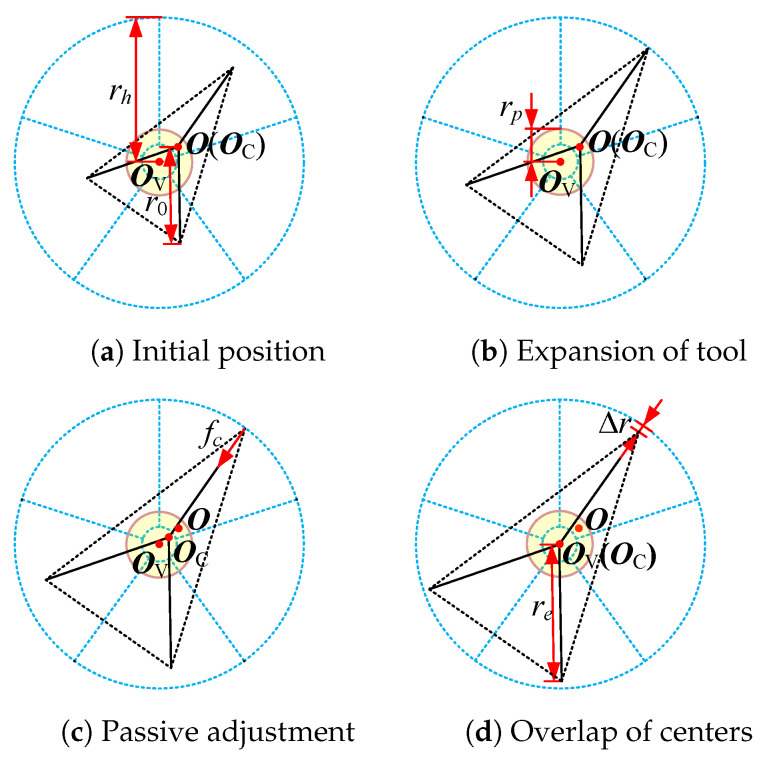
The process of achieving compliance for small deviation.

**Figure 10:**
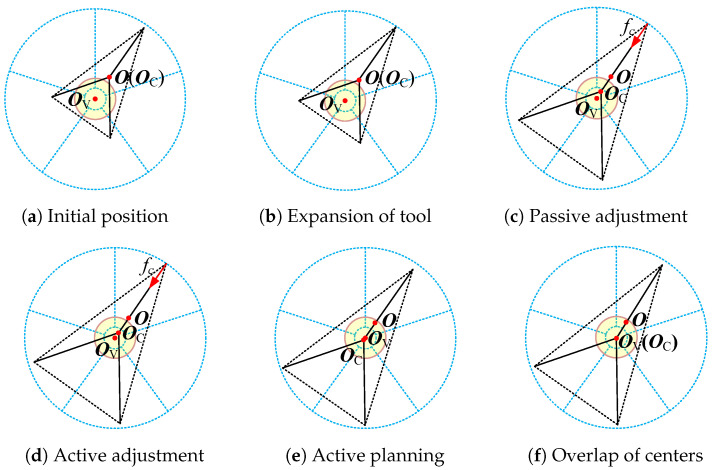
The process of achieving compliance for large deviation.

Passive compliance is advantageous for dealing with small deviations with a fast response, while active compliance ensures a large workspace. The radius of the target valve is assumed to be known a priori, with limited accuracy. The processes of achieving compliance for small and large deviations are shown in Figure 9 and Figure 10, respectively.

In Figure 9 and Figure 10, $r_{h}$ represents the radius of the valve handle; $r_{0}$ and $r_{e}$ represent the initial and final sizes of the end tool during the processes, respectively; $r_{p}$ represents the passive tolerance of the end-effector, similar to $e_{x}$ in (10); and Δr represents the final radius difference between the handle and the tool.

In Figure 9a, the blue dotted graph, black solid graph, and canary yellow region represent the five-spoke valve handle, the three-finger end tool with its initial size, and the passive tolerance, respectively. For a small deviation, O and $O_{C}$ are within the passive tolerance in their initial stages. First, the tool workspace is expanded, and after a certain amount of time, the border of the tool interacts with the border of the handle, as shown in Figure 9b.

Then, the interaction force causes point $O_{C}$ to move toward $O_{V}$, although O does not move, as shown in Figure 9c. Finally, when the valve is turned, the rotating force causes points $O_{C}$ and $O_{V}$ to coincide, as shown in Figure 9d.

In the case of a large deviation, O and $O_{C}$ are outside of the passive tolerance in the initial stages, and under this condition, hybrid passive/active compliance is applied. First, the tool workspace is expanded, as shown in Figure 10b. Second, the point $O_{C}$ moves toward $O_{V}$ owing to the passive compliance, while the point O does not move, as shown in Figure 10c. Third, active compliance control is initiated when the radial interaction force reaches its critical value, which results in both O and $O_{C}$ moving toward $O_{V}$ synchronously, as shown in Figure 10d. However, due to Δr, even when the tool expands to its maximum size $r_{e}$, $O_{C}$ cannot coincide with $O_{V}$ during the rotation of the handle. Fourth, the end-effector is controlled to move along its previous motion direction for approximately 1.5Δr, bringing O within the passive tolerance, as shown in Figure 10e. Finally, when the valve is turned, the rotating force makes points $O_{C}$ and $O_{V}$ coincide, as shown in Figure 10f.

#### 4.3.2. Algorithm for Compliant Robotic Valve-Turning Operation

In this section, the detailed algorithms for both the radial deviation and the axial handle displacement are presented.

The radial position deviation and axial displacement do not co-occur, and they can be solved via two steps. The first step explained in Algorithm 1 is to eliminate the radial position deviation, where Δr is a small user-defined parameter, $f_{x}$ and $f_{y}$ are the forces measured by the wrist F/T sensor, and $f^{1*}$ is a force boundary determining whether active compliance should be applied.

The second step illustrated in Algorithm 2, is to track the axial displacement of the handle, where $f_{z}$ is the force measured by the wrist F/T sensor, and $f^{2*}$ is a force boundary determining whether the active compliance controller should be activated.
**Algorithm 1** Hybrid passive/active compliance algorithm for radial position deviation elimination.**Prerequisite:** Position and handle radius $r_{h}$ of the target valve with limited resolution, as well as the desired force $f_{dr}$ for the radial direction.**Steps:**  1: Drive the tool fingers into the valve handle and record the tool position as $x_{0}$;  2: Expand the tool workspace: $r_{0}\to r_{e}\left(r_{e}=r_{h}-\Delta r\right)$;  3: **if** $f_{x}⩽f^{1*}$ && $f_{y}⩽f^{1*}$ **then**  4:     Passive compliance;  5: **else**  6:     Hybrid passive/active compliance;  7:     **if** $f_{x}⩾f^{1*}$ ‖ $f_{y}⩾f^{1*}$ **then**  8:         **repeat**  9:            Equation (18) activates;10:         **until** tool workspace is $r_{e}$ && $(f_{x}⩽f^{1*}$ && $f_{y}⩽f^{1*})$.11:     **end if**12:     Record the current tool position as $x_{p}$;13:     Move the tool: $l=1.5\Delta r\frac{x_{p}-x_{0}}{∥x_{p}-x_{0}∥}$;14: **end if**15: Rotate the handle through the last joint of the manipulator.

**Algorithm 2** Adaptive impedance control for axial displacement tracking.
**Prerequisite:** Desired force $f_{da}$ for the axial direction.
**Steps:**
1: Rotate the handle through the last joint of the manipulator;2: **if** $f_{z}⩽f^{2*}$ **then**3:     No compliance activates;4: **else**5:     **repeat**6:         Equation (18) activates;7:     **until** the turning operation is complete.8: **end if**


## 5. Simulation and Experiment for Robotic Valve Turning

The main factors associated with the tolerance are the two deviation angles and the axial distance between the universal joint center and the valve handle plane. The parameters of the compliant end-effector are presented in Table 3, where the maximum deflection angle $\varsigma_{1}$ can be calculated according to (4), (5), and (7). This study specifically focuses on the compliant motion of the end-effector once it has engaged with the valve handle. Positioning the end-effector close to the handle can be effectively achieved through linear interpolation [48].

It should be noted that in both the simulation and the experiment, the period from 0 to 20 s was used to move the end-effector from the vertical position to the initial position for turning the valve. From 20 to 35 s, the proposed compliant control method was implemented to bring the end-effector into the valve handle and prepare it for turning the valve. The actual valve-turning task began after 35 s. In the simulation and experimental results, to clearly present the results relevant to each stage, the starting time of some plots may vary slightly.

### 5.1. Simulation Results

**Figure 11:**
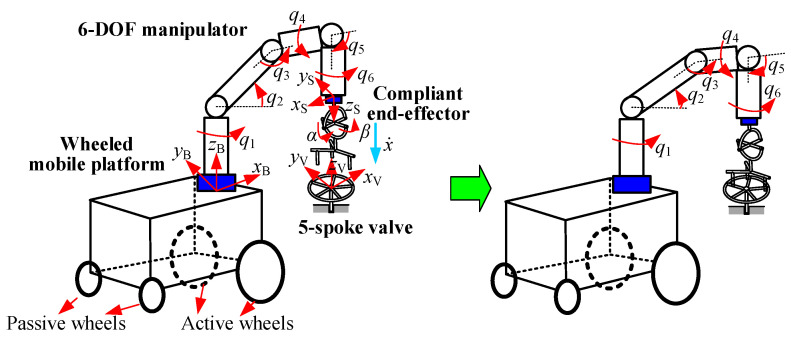
Motion of mobile manipulator during simulation for vertically installed valve.

**Figure 12:**
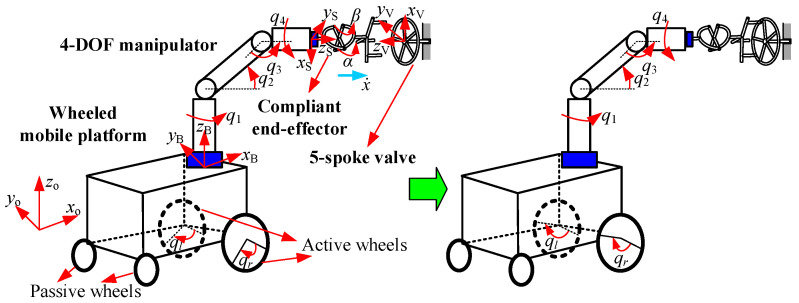
Motion of mobile manipulator during simulation for horizontally installed valve.

For active compliance, two different compliant elements to turn valves with two installation configurations were presented in Section 2. In the simulation, two conditions were employed to evaluate the valve-turning method: manipulator compliance for vertically installed valves, and hybrid platform/manipulator compliance for horizontally installed valves. Figure 11 and Figure 12 show the two simulation settings. In Figure 11, the end-effector moves vertically downward, and its trajectory *x* depends on the manipulator’s joint coordinate vector $q_{m}={\left[q_{1},q_{2},\cdots ,q_{6}\right]}^{T}$. In contrast, in Figure 12, the end-effector moves horizontally to the right, with *x* being a function of the mobile manipulator’s joint coordinate vector $q={\left[q_{r},q_{l},q_{1},q_{2},\cdots ,q_{6}\right]}^{T}$.

Three methods (no compliance, passive compliance, and hybrid passive/active compliance) under two deviation conditions (large deviation and small deviation) were compared, and the simulation parameters are presented in Table 4. The passive tolerance $r_{p}$ is determined by the point at which the terminal of the end tool contacts the handle.

The expansion of the tool workspace only occurs in the case of hybrid passive/active compliance, which aims to eliminate or reduce the radial interaction forces accompanied by $O_{C}$ coinciding with $O_{V}$, as shown in Figure 8. In all simulations, the displacement results were obtained in the valve coordinate system {V}, and the force/torque results were obtained in the sensor coordinate system {S}, as shown in Figure 11 and Figure 12.

#### 5.1.1. Simulations with Large Radial Deviation

**Figure 13:**
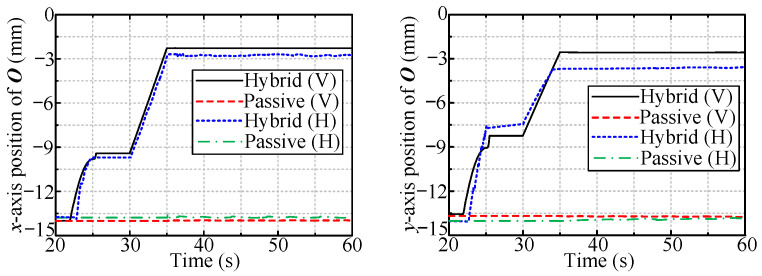
The radial displacement of O.

**Figure 14:**
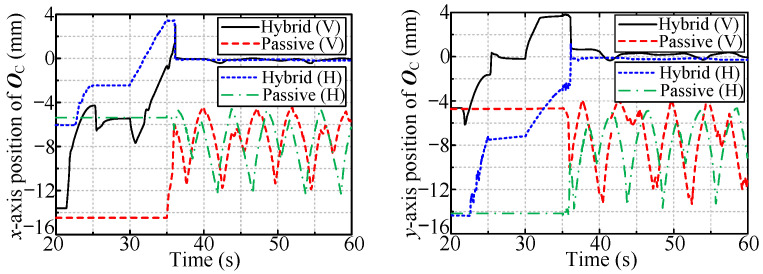
The radial displacement of $O_{C}$.

First, the simulations for large deviation (14 mm/14 mm) were conducted. Figure 13 and Figure 14 show the displacement of O and $O_{C}$ in two radial directions, respectively. The displacements of O and $O_{C}$ in the condition of no compliance are immobile in this scenario.

The tool workspace expansion occurs from 20 to 25 s, and the subsequent 5 s is reserved for stabilizing the tool. From 30 to 35 s, the tool moves in the previous direction to ensure that O is within the passive tolerance. As mentioned above, the description is for hybrid passive/active compliance; for the other two methods, the manipulator is immobile before 35 s for facilitating the comparison. For all three ways, the manipulator starts rotating the valve handle at 35 s, with a speed of 30°/s.

Figure 13 shows that the point O is immobile with passive compliance. In contrast, for the hybrid passive/active method, it moves toward the handle center due to the radial interaction force resulting from the tool workspace expansion. Figure 14 shows that only hybrid passive/active compliance can make $O_{C}$ coincide with the handle center.

**Figure 15:**
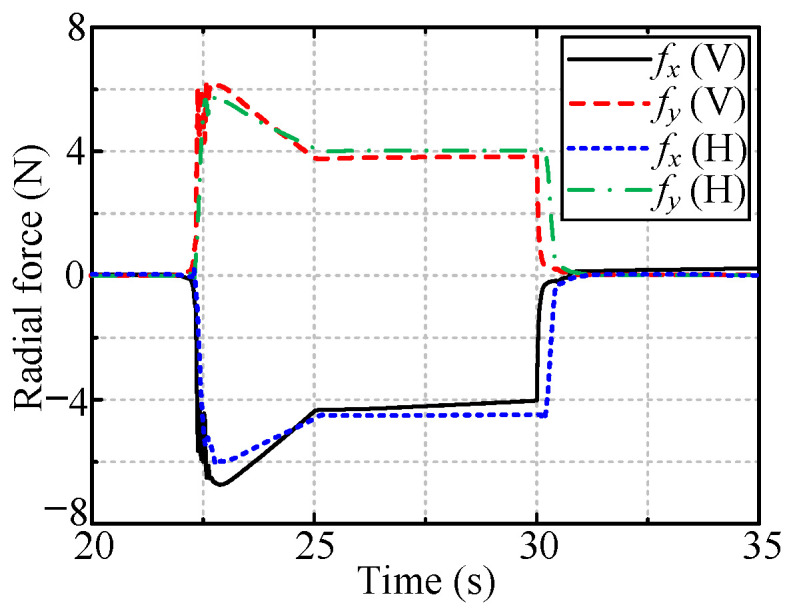
The radial forces for hybrid compliance method.

Figure 15 shows the radial interaction force during the tool workspace expansion process for the hybrid compliance method. The designed active compliance exhibits excellent performance for large radial deviation. The absolute values of all the steady-state radial forces are approximately 4 N, i.e., the desired radial force.

**Figure 16:**
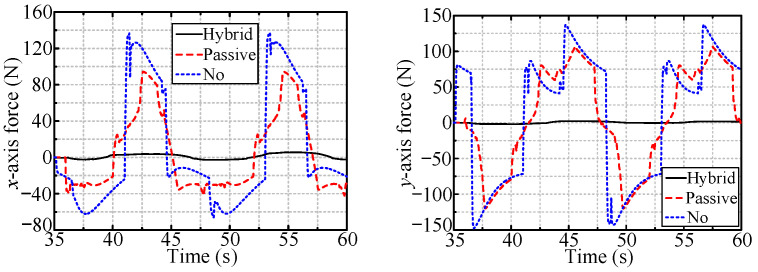
The radial forces when rotating vertically installed valve.

**Figure 17:**
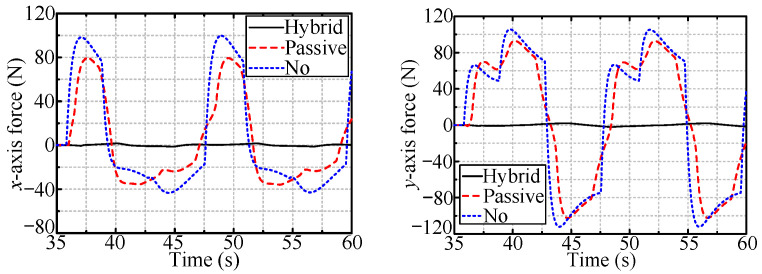
The radial forces when rotating horizontally installed valve.

Figure 16 and Figure 17 show the radial forces measured during the rotation of the handles of the vertically and horizontally installed valves, respectively. The radial forces are almost zero in the case of the hybrid compliance method and are large for the other two methods.

#### 5.1.2. Simulations with Small Radial Deviation

**Figure 18:**
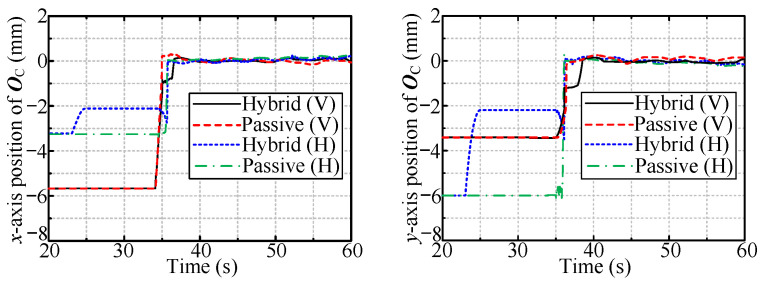
The radial displacement of $O_{C}$.

The simulation results for the case of a small radial deviation (6 mm/6 mm) are as follows. Figure 18 shows the displacement of $O_{C}$ in two radial directions. Passive compliance is enough to handle the small radial deviation; thus, O is immobile for all three compliance methods and is not shown here. $O_{C}$ coincides with the handle center $O_{V}$ for both hybrid and passive compliance, as shown in Figure 18.

**Figure 19:**
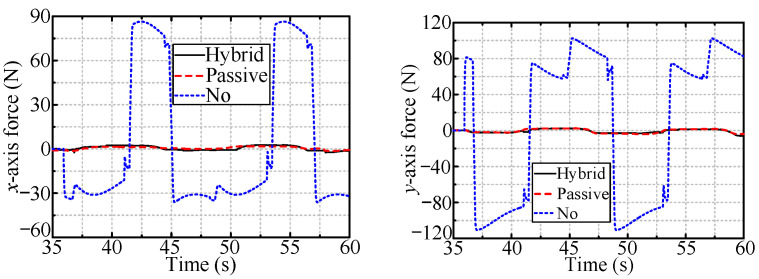
The radial forces when dealing with vertically installed valve.

**Figure 20:**
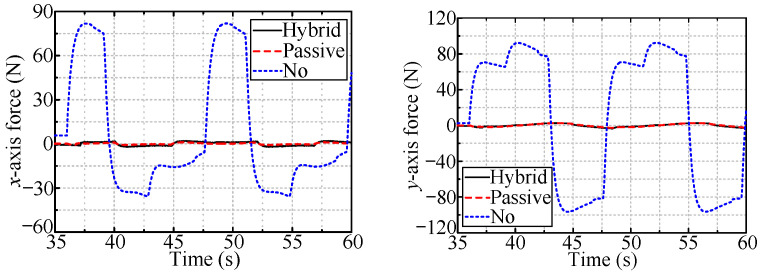
The radial forces when dealing with horizontally installed valve.

Figure 19 and Figure 20 show the radial forces for the cases of the vertically and horizontally installed valves, respectively. The radial forces are very small (close to zero) for both passive and hybrid compliance.

Next, the effectiveness of the proposed method for tracking the axial displacement of the handle was verified.

**Figure 21:**
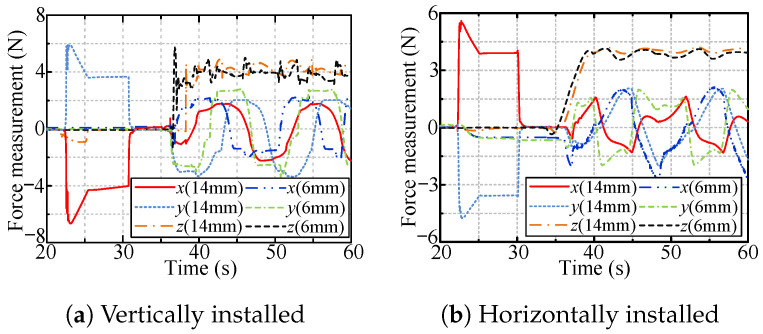
The measured forces with hybrid compliance to valves with different installation types.

Figure 21 shows the forces for robotic valve-turning with hybrid passive/active compliance for radial deviation and adaptive impedance control for axial displacement. The axial forces are all approximately 4 N, i.e., the desired axial force, indicating the effectiveness of the adaptive impedance control for tracking the handle’s axial displacement. Additionally, the small radial forces that arose during the rotation of the valve indicate the feasibility of the proposed hybrid passive/active compliance for eliminating radial position deviation.

In the simulations, hybrid passive/active compliance was most effective for robotic valve-turning among the three compliance methods. The radial position deviation was effectively eliminated, with a radial force no larger than 4 N during the valve handle rotation. This force is far smaller than the radial force that occurs when no compliance method is used (more significant than 60 N). Additionally, the axial force was close to 4 N, i.e., the desired axial force, with only approximately 10% force variation, indicating that the adaptive impedance control can effectively track the axial displacement of the valve handle.

### 5.2. Experimental Results

**Figure 22:**
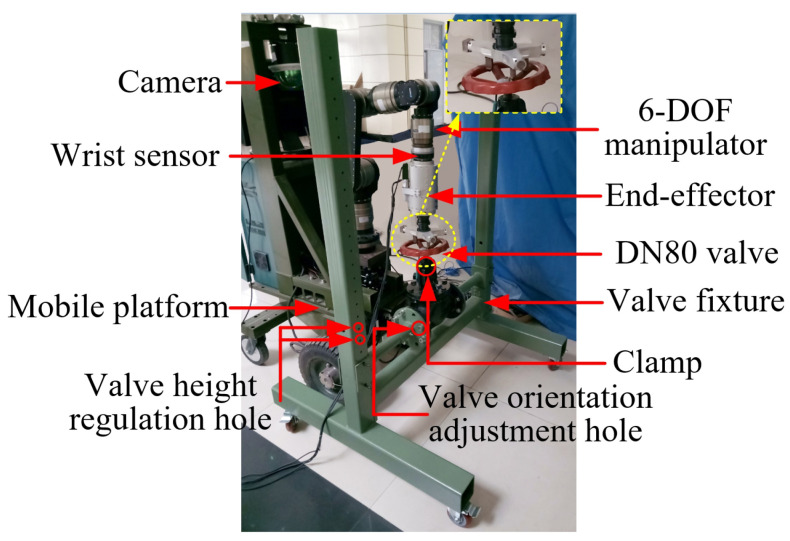
Experimental platform of robotic valve turning.

At this stage, a real-life WMM is used for the valve turning experiments (see Figure 22) [9]. It consists of a 6-DOF manipulator from SCHUNK LWA4/SDH, and a four-wheel mobile platform with two active wheels (front) and two passive wheels (back). We experimentally validated the proposed method with a vertically installed valve using manipulator compliance, similar to the first simulation. A real industrial valve (DN80) installed in a valve fixture with adjustable height and orientation was used to simulate different valves for a nuclear plant. The valve resistance torque was adjusted by tightening or releasing a clamp on the valve. The parameters of the valve handle are presented in Table 4.

**Figure 23:**
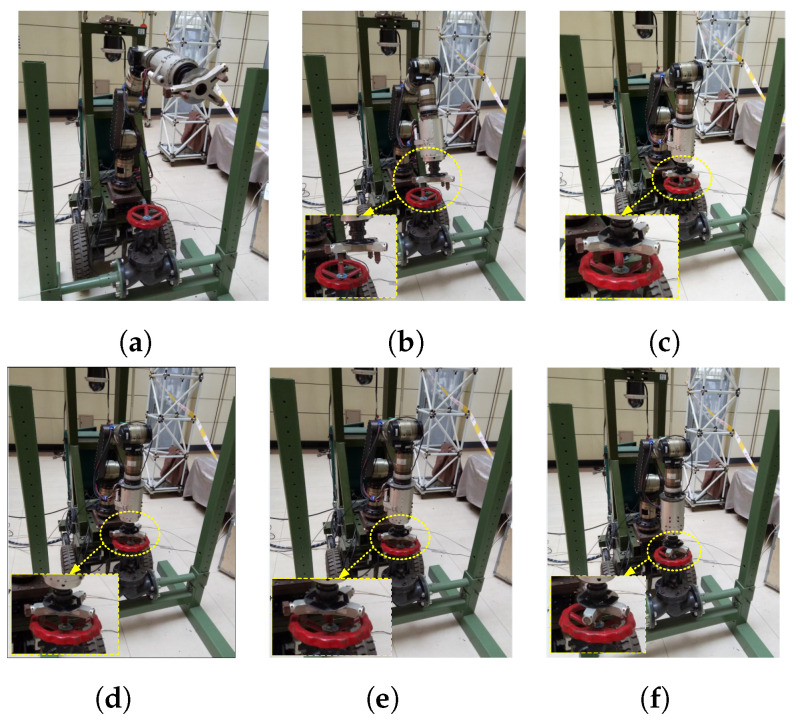
Pictures of the mobile manipulator turning the valve. Pictures (**a**–**c**) show the end-effector moves from its start position to the top of the valve with vertical orientation, (**d**) shows the end-effector moves vertically into the valve handle, (**e**) shows the end tool expands its size to eliminate radial deviation, (**f**) shows the manipulator turns the valve while tracking the axial displacement of the handle.

The height of the valve was selected within the robot workspace. The valve resistance torque was set as 6 Nm, which is smaller than that of most industrial valves, owing to the restriction of our experimental platform. The mobile manipulator was positioned in front of the valve via trajectory planning of the mobile platform, and then the platform remained immobile. The trajectory for the end-effector to enter the valve handle was pre-calculated according to the handle position obtained through a visual system with limited accuracy. Subsequently, the proposed hybrid passive/active compliance method for valve-turning was applied. Several experiments were performed to confirm the effectiveness of the proposed method compared with the passive compliance method. The controller parameter vectors for the experiments are $K_{p}=\left[2,2,4\right]$, $K_{d}=\left[0.3,0.3,0.5\right]$, $K_{i}=\left[5,5,6.4\right]$. The sequential pictures in Figure 23 illustrate the valve-turning process. The signals provided by the six-axis wrist force/torque sensor were recorded during the process.

**Figure 24:**
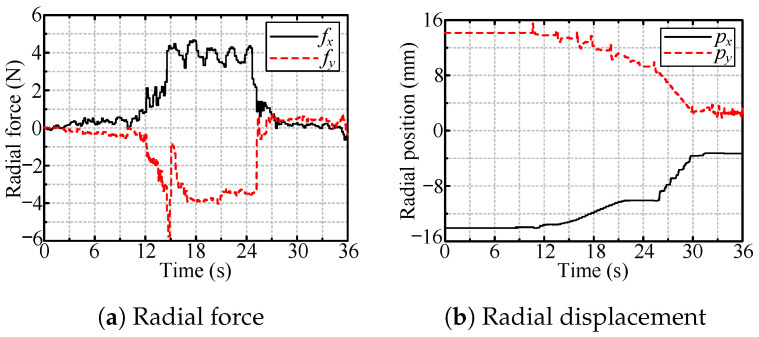
Radial force and tool position during tool workspace expansion process.

Figure 24 shows the radial force and position of the tool when the tool increased its size with a large radial deviation. The size expansion occurred from 10 s (60 mm) to 25 s (78 mm). The period of 25–30 s was employed for line motion, similar to the simulation. Figure 24a shows that the absolute value of the radial force was approximately 4 N, which was the desired radial force, and the corresponding position change in this process is shown in Figure 24b.

**Figure 25:**
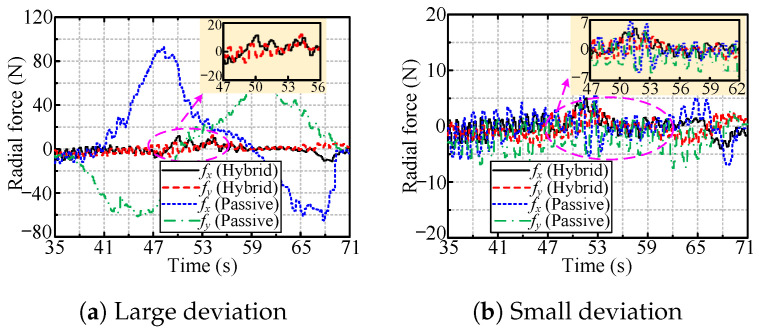
The radial force during valve-turning under different deviations.

Figure 25 shows the radial force for different compliance methods and different deviations. The radial force for a large deviation is shown in Figure 25a. In this condition, the hybrid compliance method was significantly better than the passive compliance method, with a maximum radial force of only approximately 9 N. The radial force for small deviation is shown in Figure 25b. In this condition, the two methods exhibited very similar results owing to the effectiveness of passive compliance.

**Figure 26:**
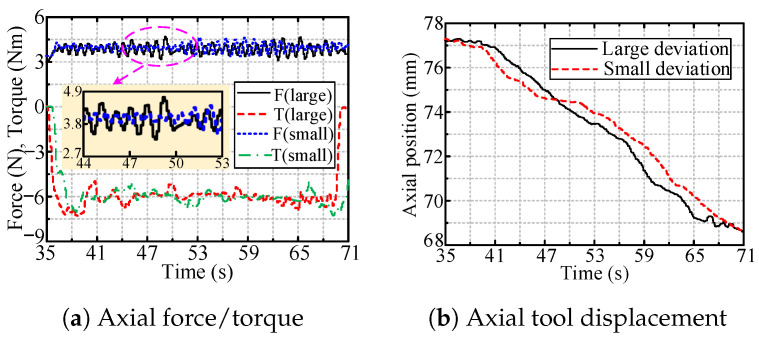
The axial force/torque and tool displacement during the handle axial position tracking process.

Figure 26 shows the axial force/torque and tool displacement that occurred during the tracking of the axial handle position. Figure 26a shows that for both large and small deviations, the axial force was approximately 4 N, following the desired force, and the rotating torque was around 6 Nm, which was the valve resistance torque. Figure 26b shows the corresponding tool displacement in this process, which was approximately 8 mm.

The experimental results indicate the effectiveness of the proposed method for valve-turning. A comparison of hybrid passive/active compliance with passive compliance reveals the following results. First, in cases of large radial deviations, the radial rotating force decreased from approximately 60 N with passive compliance to 9 N with hybrid passive/active compliance. Second, the high tracking performance of the proposed method was demonstrated by the axial force, which was approximately 4 N with only about 13% variation.

This paper focuses on achieving compliant interaction between the end-effector and the valve handle after the end-effector makes contact, ensuring no excessive contact force is generated during the valve turning process. In teleoperation applications, this process can be enhanced by integrating computer vision for recognizing the relative pose of the valve and employing artificial intelligence for teaching and learning the valve turning operation. This integration enhances the autonomy of valve turning while ensuring compliant operation. Additionally, enabling a WMM to operate in extreme environments and approach the valve is crucial for completing a valve turning task. In this process, artificial intelligence-based navigation and motion planning, combined with computer vision, play an essential role.

## 6. Conclusions

A hybrid passive/active compliance method is developed for robotic valve-turning towards rescue operations in this paper, along with experimental results. The main conclusions are as follows:

(1) A hybrid passive/active compliance method was proposed. The passive compliance is realized by a novel detachable compliant end-effector based on a three parallel universal joint mechanism, which can eliminate approximately 3.5° of the angular deviation and 9.7 mm of the position deviation in each radial direction. The active compliance is achieved by a position-varying tracking controller that performs adaptive impedance control.
(2) A specific method for valve-turning was presented. Hybrid passive/active compliance is used for the radial position deviation, active compliance is used for the axial displacement of the valve handle, and a detachable end-effector is used for valve handles with different types and sizes.
(3) Robotic valve-turning simulations for vertically and horizontally installed valves and experiments for a horizontally installed valve were conducted. The results indicated that the proposed hybrid passive/active compliance can reduce the radial rotating force by more than 85% compared to passive compliance. Additionally, the adaptive impedance control can effectively track the desired axial force, with only approximately 13% force variation.

Our future research aims to develop a stereo vision system capable of detecting valves and identifying their sizes. Additionally, we plan to explore the replacement of tools for turning valves with those designed for various handle types. Furthermore, we will consider incorporating artificial intelligence to enable autonomous valve turning.

## Figures and Tables

**Table 1:** Combination of compliance methods to problems (A) and (B).

Deviation Type						Possibility
Δx	Δy	Δz	Δφ	Δθ	Δψ	
A	A	A	A	A	/	✔
P	P	A	P	P	/	✔
A + P	A + P	A	A + P	A + P	/	✔✔✔
A	A	P	A	A	/	✘
A	A	A + P	A	A	/	✘
P	P	P	P	P	/	✘
P	P	A + P	P	P	/	✘
A + P	A + P	P	A + P	A + P	/	✘
A + P	A + P	A + P	A + P	A + P	/	✘

“A” represents “active compliance”, and “P” represents “passive compliance”.

**Table 2:** Analysis of compliant elements for active compliance.

Valve Installation	Compliant Elements	Fewest No. of Actuators	Specific Response
Vertical	MP + M	6	MP→ (A)
			MP & M → (B)
	M	8	M → (A)
			M → (B)
Horizontal	MP + M	6	MP & M→ (A)
			MP → (B)
	M	8	M → (A)
			M → (B)

“MP” represents “mobile platform”, “M” represents “manipulator”, and the fewest number of actuators include the actuators of the mobile platform, which are 2 in our mobile system, and the manipulator.

**Table 3:** Design parameters of compliant end-effector.

Parameter	Description	Value
$r_{1}$	radius of the input rod	50 mm
$r_{2}$	radius of the output rod	49 mm
$r_{3}$	shaft radius of the input rod	5 mm
$r_{4}$	hole radius of the output rod	10 mm
$d_{1}$	forward width of the output rod	12 mm
$d_{2}$	backward width of the output rod	18 mm
$\varsigma_{1}$	maximum deflection angle	3.5°
$l_{o}$	length of the output rod	160 mm

**Table 4:** Control parameters during the simulation.

Parameter	Description	Value
$r_{h}$	radius of valve handle	80 mm
$p_{h}$	pitch of valve handle	3 mm
$\tau_{h}$	resistance torque of valve	6 Nm
$r_{0}$	initial radius of end tool	60 mm
$r_{e}$	final radius of end tool	78 mm
Δr	radius difference	2 mm
$r_{p}$	passive tolerance	9.7 mm
$f_{dx}/f_{dy}/f_{dz}$	desired forces	4 N/4 N/4 N
$K_{p}/K_{d}/K_{i}$	control parameters in (18)	[8, 8, 10]/[1.2, 1.2, 1]/[10, 10, 14]

## Data Availability

Data are contained within the article.
